# Contemporary procedure characteristics and outcomes of accessory atrioventricular pathway ablations in an integrated community-based health care system using a tiered approach

**DOI:** 10.1186/s12872-021-02132-0

**Published:** 2021-06-30

**Authors:** Charlie Young, Annie Kwan, Lisa Yepez, Meghan McCarty, Amanda Chan, Dora Hsu, Jennifer Han, Taresh Taneja, Shirley Park, Robert Hayward, Taylor I. Liu

**Affiliations:** Department of Cardiac Electrophysiology, Kaiser Permanente NCAL, Kaiser Santa Clara Medical Center, 710 Lawrence Expressway, Department 342, Santa Clara, CA 95051 USA

**Keywords:** Radiofrequency ablation, Accessory pathway ablation outcome, Wolff Parkinson White Syndrome

## Abstract

**Background:**

Since the early descriptions of large series of accessory atrioventricular pathway ablations in adults and adolescents over 20 years ago, there have been limited published reports based on more recent experiences of large referral centers. We aimed to characterize accessory pathway distribution and features in a large community-based population that influence ablation outcomes using a tiered approach to ablation.

**Methods:**

Retrospective analysis of 289 patients (age 14–81) who underwent accessory ablation from 2015–2019 was performed. Pathways were categorized into anteroseptal, left freewall, posteroseptal, and right freewall locations. We analyzed patient and pathway features to identify factors associated with prolonged procedure time parameters.

**Results:**

Initial ablation success rate was 94.7% with long-term success rate of 93.4% and median follow-up of 931 days. Accessory pathways were in left freewall (61.6%), posteroseptal (24.6%), right freewall (9.6%), and anteroseptal (4.3%) locations. Procedure outcome was dependent on pathway location. Acute success was highest for left freewall pathways (97.1%) with lowest case times (144 ± 68 min) and fluoroscopy times (15 ± 19 min). Longest procedure time parameters were seen with anteroseptal, left anterolateral, epicardial-coronary sinus, and right anterolateral pathway ablations.

**Conclusions:**

In this community-based adult and adolescent population, majority of the accessory pathways are in the left freewall and posteroseptal region and tend to be more easily ablated. A tiered approach with initial use of standard ablation equipment before the deployment of more advance tools, such as irrigated tips and 3D mapping, is cost effective without sacrificing overall efficacy.

**Supplementary Information:**

The online version contains supplementary material available at 10.1186/s12872-021-02132-0.

## Introduction

Catheter ablation using radiofrequency (RF) energy has been the cornerstone of therapy for patients with accessory atrioventricular pathways (AP) since its safety and efficacy was first described in the 1990s [1–5]. These reports demonstrated high acute success and low complication rates. Early reports have also identified factors associated with failure of ablation procedures. Failure of procedures have been attributed to technical challenges such as poor catheter stability, failure to record AP potential, and pathway locations such as right sided, posteroseptal, and anteroseptal regions [6, 7]. Reasons for prolonged or failed attempts at radiofrequency catheter ablation of accessory pathways were primarily due to difficulties in catheter manipulation and inaccurate mapping [8]. These studies included short term follow up and have not provided important procedure characteristics such as fluoroscopy time and case times which are crucial in the procedure planning process. Furthermore, these studies do not reflect more recent experience with the use of newer technologies such as irrigated catheters and 3D electroanatomic mapping. A recent study in the pediatric population examined long-term outcomes of AP catheter ablation and identified pathway features that predict ablation failure or long-term recurrence [9]. Comprehensive assessment of patient and AP features that influence procedural time parameters, acute, and long-term success has not been described in the adolescent and adult population in the modern era.

In this study, we examined our institution’s long-term experience using a tiered approach that starts with standard ablation equipment and catheter manipulation techniques. 3D mapping, open irrigated ablation, or transeptal access are considered to be “second tiered”, or supplemental and were deployed during the procedure only when needed at the discretion of the operator.

## Methods

### Patient selection and follow up protocol

We identified 289 consecutive patients ranging in age from 14 to 81, who underwent RF ablation procedures for accessory AP from 2015 to 2019 at Kaiser Medical Center, Santa Clara, California, USA. The source population included the Kaiser Permanente Northern California Heath Care System with more than 4 million members with demographics reflective of the US population [10]. Cardiac electrophysiology services for the large and diverse population are centralized at one location within the healthcare system which reduced selection bias based on geographic location. Patients included those with (1) ventricular pre-excitation with documentation of SVT or atrial fibrillation, (2) asymptomatic ventricular pre-excitation, or (3) SVT with concealed accessory AP.

A 12-lead ECG was obtained immediately post ablation and at least one-year post ablation in all patients. Patient charts were reviewed at 1–5 years post ablation. Recurrences were identified by return of ventricular pre-excitation or recurrence of documented SVT by ECG or event monitor at any point during follow up period. Intermittent pre-excitation was categorized as recurrence. All clinical encounters were reviewed within the follow up period for recurrence of ventricular pre-excitation or SVT. Patients with less than one year follow up were excluded. The study protocol including ethics review was approved by the Institutional Review Board of Kaiser Permanente.

### Ablation procedure

During comprehensive electrophysiology study, recordings from the tricuspid and mitral annulus were obtained using 7 French steerable duodecapolar or decapolar catheters inserted into the coronary sinus. Recordings were also obtained from the His bundle as well as the right ventricular apex using 5–6 French quadripolar catheters. Mapping and ablation were performed using standard 4–5 mm closed tip ablation catheters. Open-irrigated catheters, remote magnetic navigation (Stereotaxis, St. Louis, MO), and 3D mapping with Carto (Biosense Webster, Diamond Bar, CA) or Ensite (Abbott, Santa Clara, CA) mapping systems were used at the operator’s discretion. In general, our institutional preference for initial ablation procedure is to use standard non-irrigated ablation catheters without 3D mapping. For left sided APs, our initial approach is generally retrograde transaortic rather than via transeptal puncture. The retrograde transaortic approach in our experience is an established approach that is both safe and effective, and requires less equipment, and is generally less cumbersome to deploy. Use of transseptal approach is at the discretion of the operator. If transseptal approach was done, Brockenbrough needle was used with intracardiac echocardiography guidance. Irrigated catheters were used in 11% of initial ablations and 36.4% of repeat ablation procedures. 3D mapping was used in 3.9% of initial procedures and 31.8% of repeat procedures (Tables 1, 2).

**Table 1:** Patient characteristics

Patient and procedure characteristics	All Patients (N = 281)	Anteroseptal (N = 12)	Left freewall (N = 173)	Posteroseptal (N = 69)	Right freewall (N = 27)	*p* value
Female	117 (41.6%)	4 (33.3%)	70 (40.5%)	30 (43.5%)	13 (48.1%)	ns
Age (years)	38 ± 27	28 ± 22	39 ± 25	37 ± 30	32 ± 21	ns
LVEF (%)	60 ± 5	60 ± 5	60 ± 5	60 ± 5	60 ± 5	ns
Manifest pathway	116 (41.3%)	7 (58.3%)	55 (31.8%)	36 (52.2%)	15 (55.6%)	***0.004***
Multiple pathways	10 (3.6%)	0 (0%)	4 (2.3%)	3 (4.3%)	3 (11.1%)	ns
Secondary arrhythmia	11 (3.9%)	1 (8.3%)	5 (2.9%)	4 (5.8%)	1 (3.7%)	ns
Tachyarrhythmia:						
AF	18 (6.4%)	0 (0%)	9 (5.2%)	6 (6.9%)	3 (11.1%)	ns
AVRT	227 (80.8%)	11 (91.7%)	149 (86.1%)	44 (63.8%)	23 (85.2%)	< ***0.001***
Open Irrigated Ablation	31 (11.0%)	1 (8.3%)	13 (7.5%)	15 (21.7%)	2 (7.4%)	***0.014***
3D Mapping	11 (3.9%)	2 (16.7%)	4 (2.4%)	4 (5.8%)	1 (3.7%)	ns
Fluoroscopy Time (min)	17 ± 22	33 ± 30	15 ± 19	19 ± 24	19 ± 26	< ***0.001***
Ablation Application Duration (sec)	278 ± 330	422 ± 318	278 ± 398	270 ± 289	519 ± 539	***0.003***
Total Case Time (min)	155 ± 74	203 ± 77	144 ± 68	160 ± 75	155 ± 96	< ***0.001***
Acute Procedure Success	266 (94.7%)	9 (75.0%)	168 (97.1%)	66 (95.7%)	23 (85.2%)	***0.001***
Long term Success After Initial Acute Success	248 (93.2%)	7 (77.8%)	165 (98.2%)	60 (91.0%)	21 (91.3%)	< ***0.001***

**Table 2:** Repeat procedures

	Repeat procedures (N = 22)
Anterior/anteroseptal AP	2 (9.1%)
Left freewall AP	8 (36.4%)
Posteroseptal AP	7 (31.8%)
Right freewall AP	5 (22.7%)
First procedure at outside facility	8 (36.4%)
Open irrigated ablation	8 (36.4%)
3D mapping	7 (31.8%)
Acute success	21 (95.5%)
Long term success	21/21 (100%)

Non-irrigated RF ablations were performed in temperature-controlled mode targeting 30–50 W with temperature limit of 55–70 °C. Open-irrigated ablations were performed targeting 20–45 W depending on the location of the AP. Power output was limited to ≤ 25 W for ablations inside the coronary sinus. Ablations were performed in 30–60 s intervals with end point of anterograde and retrograde AP conduction block and SVT non-inducibility. Post ablation, if acutely successful, at least 30 min of wait period was generally included prior to termination of procedure.

### Accessory pathway characteristics and ablation outcome analysis

AP ablation locations were separated into four anatomic groups, anteroseptal (AS), left freewall (LFW), posteroseptal (PS), and right freewall (RFW), as previously described [11]. PS region in this study encompass all septal pathways, including mid-septal from below the His to the coronary sinus ostium and is separated into three regions, left posteroseptal (LPS), right posteroseptal (RPS), and subepicardial coronary venous system (Epi-CS). The AS group included both true para-Hisian pathways defined by the presence of a His potential recorded at the site of ablation, and right anterior pathways located within one centimeter of the His recording site as viewed in LAO 45°.

### Prolonged ablation times

The total procedure, fluoroscopy exposure, and ablation energy application times were recorded in all cases. Prolonged times were defined as follows: (1) total procedure time greater than 200 min, (2) fluoroscopy exposure time greater 30 min, and (3) ablation energy application time ≥ 400 s.

### Statistics

Statistical analysis was performed using Prism 8.4.2 software (GraphPad Software, San Diego, CA) and Microsoft Excel for Office 365 (Microsoft Corporation, Redmond, WA). Numerical data are presented as median ± interquartile range. For continuous variables, comparisons among groups were analyzed using one-way ANOVA test. To assess statistical significance of differences in frequency of dichotomous variables, contingency tables were created comparing observed frequencies to expected frequencies using Chi-square test. *p* values are presented for all statistical analyses.

To compare freedom from recurrence post ablation procedures, survival data was plotted using the Kaplan–Meier method. The log rank test was used to detect statistical difference among groups.

## Results

### Patient and accessory pathway characteristics

**Fig. 1:**
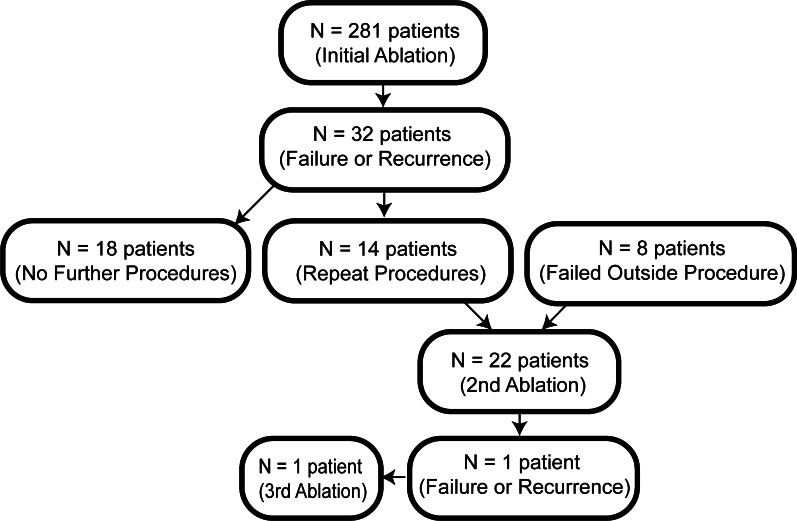
Study patients. Number of patients in our cohort

During the study period (2015–2019), a total of 304 accessory AP ablation were performed. 281 of these procedures were initial ablations (Fig. 1). 22 patients underwent repeat ablation which were all successful without long term recurrence except for one, for whom a third procedure was performed successfully.

**Fig. 2:**
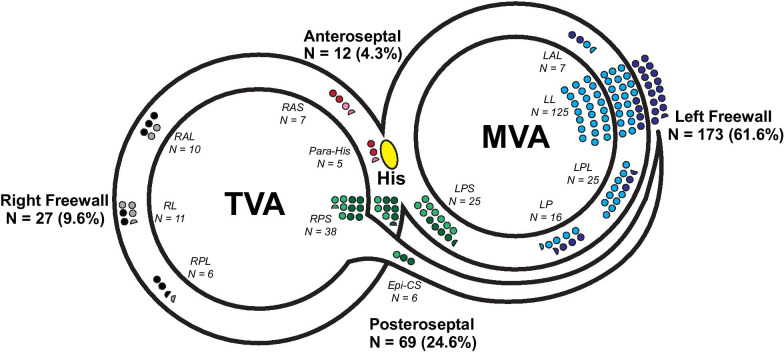
Distribution of accessory atrioventricular pathways. The number and proportion of patients with accessory AP locations are displayed along tricuspid valve annulus (TVA) and mitral valve annulus (MVA). His bundle recording is labeled yellow. Number of patients are proportionately represented by the number of circles (each circle represents 2 patients). Anteroseptal pathways are red (manifest) or pink (concealed). Left freewall pathways are dark blue (manifest) or light blue (concealed). Posteroseptal pathways are dark green (manifest) or light green (concealed). Right freewall pathways are black (manifest) or gray (concealed). Sub-regional locations are listed. *Epi-CS* epicardial-coronary sinus, *LAL* left anterolateral, *LL* left lateral, *LP* left posterior, *LPL* left posterolateral, *LPS* left posteroseptal, *RAL* right anterolateral, *RAS* right anteroseptal, *RL* right lateral, *RP* right posterior, *RPL* right posterolateral, *RPS* right posteroseptal

The distribution of pathway locations was not uniform (Fig. 2). Two groups together accounted for 83.2% of cases (LFW: 61.6% and PS: 24.6%). LFW pathways were more likely to be concealed (68.2%) compared to pathways from other locations (*p* = 0.004; Table 1). RFW and AS pathways were relatively uncommon comprising 8.2%, and 5.7% of all cases respectively.

In our population, 80.8% of patients undergoing RF ablation had demonstrated atrioventricular reciprocating tachycardia (AVRT). Multiple accessory pathways were reported in 3.6% of cases (Table 1).

Patient characteristics such as age, sex, and left ventricular function were not statistically different among the four AP groups (Table 1).

Major complications included two cases of AV block requiring pacemaker implants (one from LPL group and one from RPS group). One patient had a femoral arterial pseudoaneurysm (LPL group). Other known major complications such as cerebral vascular events, cardiac perforation, tamponade, myocardial infarction or death did not occur.

### Initial procedure acute outcome

For left atrial and ventricular access, the initial approach was retrograde transaortic (196 of 199 cases, 98.5%), with 173 of these cases being LFW pathway ablations. Overall use of open-irrigated catheters and 3D mapping during initial ablation was 11% and 3.9%, which was much lower than during repeat ablations (36.4% and 31.8%, respectively). More open-irrigated catheters were used in the PS group (21.7%) with 7/15 of these cases being inside the coronary sinus system (Table 1).

**Fig. 3:**
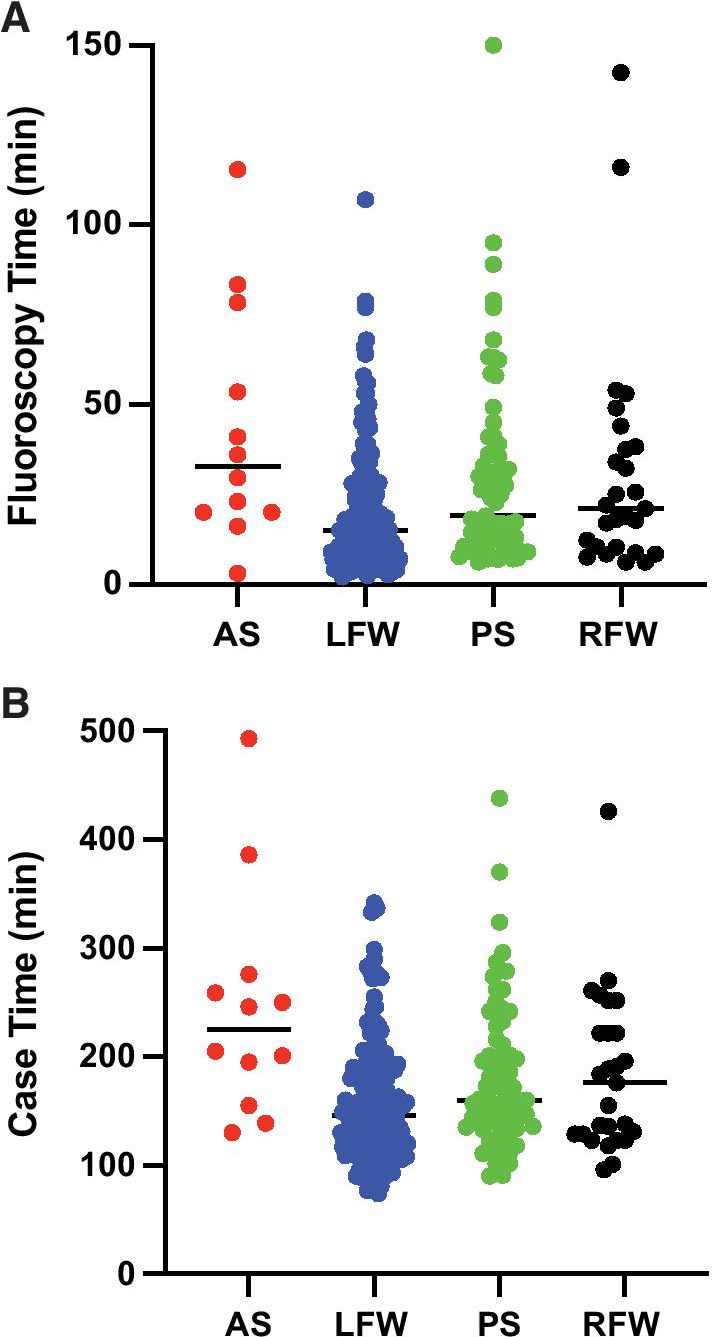
Case times and fluoroscopy times. The total case times (**a**) and fluoroscopy times (**b**) for the initial ablation procedures are shown for each AP location group, anteroseptal (red), left freewall (blue), posteroseptal (green), and right freewall (black). Each dot represents one case. Median times are also displayed as black horizontal line

Acute success was achieved in 94.7% of all initial procedures (LFW: 97.1%; PS: 95.7%; RFW: 87%; and AS: 75%) (Table 1). Median fluoroscopy time was highest in AS group (33 ± 30 min) and lowest in LFW group (15 ± 19 min). Longest ablation duration times were in AS (422 ± 318 s) and RFW (519 ± 539 s) groups. Total case time as defined by time from patient arrival to exit from the EP laboratory was longest in AS group (203 ± 77 min) and shortest in LFW group (144 ± 68 min) (Table 1 and Fig. 3). Significant number of outliers were seen in each group (Fig. 3).

### Repeat procedure acute and long-term outcome

As part of our tiered approach, 3D mapping and open irrigated ablations were more commonly used for failed initial procedure whether the initial attempt was performed at our institution or at another institution. Open irrigated ablation was used in 36.4% and 3D mapping was used in 31.8% of repeat procedures. Long term success of all repeat procedures was 100% (Table 2).

### Procedural efficiency

The absolute number and proportion of cases with prolonged procedure time parameters (fluoroscopy, ablation, and case times) for all four groups were tabulated (Table 3). Anatomic locations that had a greater than 50% of cases with prolonged time parameters were underlined. These locations consisted of AS, LAL, Epi-CS, and RAL. Common reasons cited for prolonged times were, (1) limited mapping capability, (2) limited lesion formation, (3) difficult ablation catheter navigation, (4) multiple chambers mapped, and (5) incidental secondary arrhythmias requiring ablation.

**Table 3:** Cases with prolonged procedure, fluoroscopy, and ablation times

	Procedure time ≥ 200 min	Fluoroscopy time ≥ 30 min	Ablation time ≥ 400 s
All Patients (N = 281)	66 (23.5%)	76 (27.0%)	88 (31.3%)
Anteroseptal (N = 12)	**8 (66.7%)**	**6 (50.0%)**	**7 (58.3%)**
Left Freewall (N = 173)	29 (16.8%)	35 (20.2%)	41 (23.7%)
Left AL FW (N = 7)	***6 (85.7%)***	***5 (71.4%)***	***4 (57.1%)***
Posteroseptal (N = 69)	20 (29.0%)	25 (36.2%)	23 (33.3%)
Right PS (N = 38)	*11 (28.9%)*	*12 (31.6%)*	*13 (34.2%)*
Left PS (N = 25)	*6 (24.0%)*	*11 (44.0%)*	*7 (28%)*
Epi-CS (N = 6)	***3 (50.0%)***	2 (33.3%)	***3 (50%)***
Right freewall (N = 27)	9 (33.3%)	10 (37.0%)	**17 (63.0%)**
Right AL FW (N = 6)	***4 (66.7%)***	***3 (50.0%)***	***3 (50.0%)***

Locations with 50% or greater prolonged time parameters are underlined and highlighted

### Long term outcome of successful initial ablation procedures

**Fig. 4:**
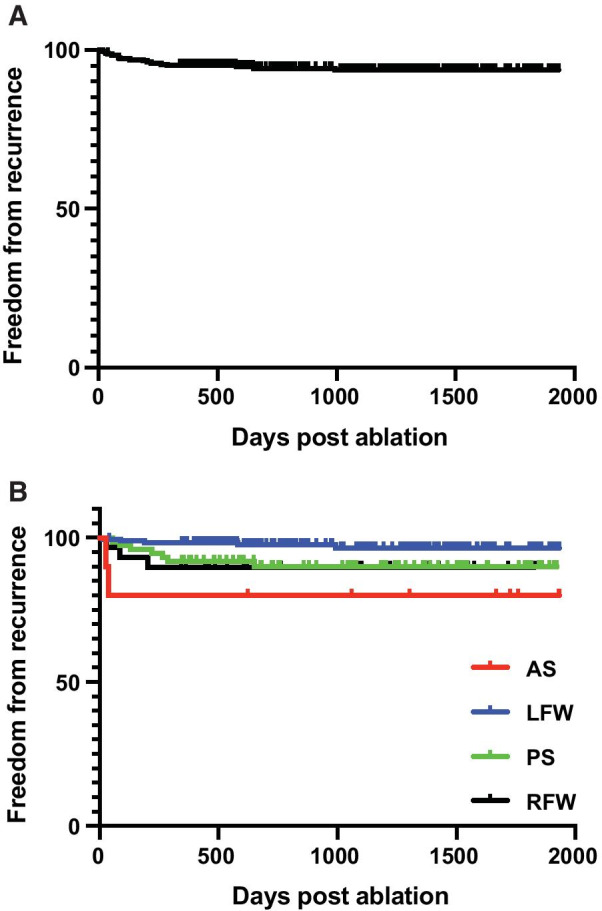
Freedom from recurrence post ablation. Kaplan Meier curve depicting the long-term freedom from recurrence of ventricular pre-excitation or SVT in all patients (**a**) and (**b**) among patients with AP at specific location groups, anteroseptal (red), left freewall (blue), posteroseptal (green), right freewall (black)

With median follow up of 931 days post ablation in patients who had initial successful ablations, 93.2% remained free from recurrence of SVT or ventricular pre-excitation (Fig. 4a). Long term success was lower at 77.8% for AS pathways compared with 98.2% for LFW pathways (*p* = 0.002 log-rank test; Fig. 4b). RFW and PS pathways have similar long-term outcome (91.0% and 91.3% respectively, Fig. 4b). There were no statistical differences by age, sex, or ventricular pre-excitation in acute or long-term outcome (data not shown).

## Discussion

Over a four-year period from 2015 through 2019, 289 patients underwent RF ablation procedure for AP in our institution. The source population included the Kaiser Permanente Northern California Health System with diverse demographics that reflect large portions of the US population. As the sole referral center for this population, 97.2% patients in this study had their first ablation at our institution. Without a referral bias towards complexity and repeat ablations, this study was able to identify all ablations derived from a large population group and was able to give an accurate picture regarding the distribution of pathway locations within the large population.

### Distribution of pathway locations

Consistent with prior studies [3, 4], we found that the most common location of accessory pathways was left freewall followed by posteroseptal region. There may be developmental correlates of this finding that is consistent in pediatric and now adolescent and adult populations. In the early stages of cardiac development, the developing left ventricle is continuous with the atrial myocardium including the basilar aspect of the eventual septum [12]. The development of accessory muscular connections may be a vestige of the atrial-LV connection that persisted through the formation of the fibroadipose insulating tissue of the atrioventricular canal and thus more common along the mitral annulus. Interestingly, we also found that, distinct from other locations, the majority of LFW pathways only demonstrated retrograde conduction. This may be due in part to the distinctive embryologic origin of the mitral annulus to the tricuspid annulus. The tricuspid annular tissue derives not only from atrioventricular canal myocardium like the mitral annulus but also primary ring tissue which forms much of the normal anterograde decremental conduction system [12]. Findings in this study and others suggest such correlation with the developing cardiac conduction system and pathophysiology of accessory atrioventricular pathways [13].

### Outcome of ablation at different locations

Our acute procedural success (94.7%) and major complication rate (1.0%) for all ablations are in line with previous large reported series [4, 14]. Acute success rates varied amongst groups with highest success for LFW pathways (97.1%) and lowest success for AS pathways (75%). Lower success rate was associated with a greater degree of difficulty as measured by prolonged procedural time parameters.

We were able to make comparisons on the degree of difficulty encountered during AP ablation between major groups as well as identify specific regions within each group that were particularly more challenging to ablate. From review of the reasons for prolonged times contained in the procedure report, we surmise that transient loss of AP conduction during ablation energy delivery was the most common cause of prolonged time parameters. Inability to create an effective and durable lesion for complete ablation of the AP may be the result of (1) poor ablation catheter contact, (2) power output limited by temperature, (3) limited proximity to actual site of pathway. These potential reasons for prolonged procedure time parameters were likely due to problematic pathway anatomic locations.

### Difficult pathway locations for ablation

Pathways located in the LAL region can be uniquely difficult as evidenced by the high proportion of cases with prolonged case times (85.7%). A potential explanation is that mapping becomes more limited by the inability to employ multipole catheter recordings effectively as the coronary sinus diverges away from the mitral valve annulus as it courses more anteriorly. Catheter stability can also be limiting in this region. As a group, Epi-CS pathways are generally more difficult as evident by a higher proportion of cases having prolonged time parameters which is in general due to need for multi-chamber mapping. RFW pathways were also challenging with a significantly lower acute success rate (87%). The angle of entry into the RA from the IVC favors the catheter to be directed towards the septum and requires additional manual torque for the catheter to reach and contact the lateral wall. These anatomical characteristics makes catheter maneuverability and stability more difficult, as reflected by the significantly longer ablation application time required in this group. AS pathways presented challenges due to its proximity to the AV node. Although risk of AV node injury is minimized with ablation approached from the ventricular side [4], maneuvering the catheter to a stable position in the right ventricle is inherently difficult and accounts for highest total case times among the four groups.

In situations where a difficult pathway is encountered at certain anatomic locations, supplemental tools may provide the specific solution needed to achieve a successful outcome. This is supported by the fact that open irrigated catheters were required to achieve success more frequently during the initial attempt of right posteroseptal pathways (21.7%) in which 7/15 of these cases being for ablation inside the coronary sinus system (Table 1). Furthermore, there was a greater use of irrigated catheters (36.4% vs. 11%) and 3D mapping (31.8% vs. 3.9%) on successful repeat procedures compared to the initial failed attempt.

In our experience, 3D mapping enhances mapping accuracy in cases where multipolar mapping is not ideal such as in LAL and RAL locations. Open-irrigated ablation can also ensure more effective lesions inside the CS vein and during ablation on the ventricular side of the myocardium where close tissue contact results in limited power output limited by temperature elevations. Where available, remote magnetic navigation (Stereotaxis) may enhance maneuverability of the ablation catheter when targeting specific pathway locations [15].

### Efficiency and cost saving

Although use of additional tools beyond the initial standard approach can be critical for success in difficult cases at specific anatomic locations, the equipment and supplies are more costly and require increased set up time and staffing. In our opinion, a more judicious approach to their use only in cases where the specific need is matched by the solution offered by the tool is both more procedural and cost efficient. In our practice, this has been a long-established preference supported by findings in this report that the majority of accessory pathways, particularly those on the left free wall of the mitral annulus and right posteroseptal region along the tricuspid annulus, are easily mapped and successfully ablated with the usual standard equipment and techniques. In addition, the indiscriminate use of irrigated catheters that do not have the inherent safety of temperature-controlled ablation potentially adds unnecessary risk in a majority of cases where their use does not directly affect outcome. Based on these reasons, we prefer to refrain from unnecessary use of more costly equipment that in addition, require more staffing and time. As a result, our overall use of open-irrigated (11%) and 3D-mapping (3.9%) catheters at the initial ablation procedure was much lower compared to other studies [9, 16, 17].

We recognize that this goes against current trend in the practice community which is towards greater reliance of transseptal access for left sided cases and the deployment of 3D mapping system and irrigated ablation catheters as the default. However, we have demonstrated that most AP ablations could be routinely performed successfully and efficiently with standard equipment and approach.

We estimated the typical actual cost per case accounting for utilization of disposable equipment in our lab in 2021 US dollars. The typical comparative acquisition cost of disposable supplies per case for standard equipment with retrograde aortic access for left sided APs versus 3D mapping and irrigated ablation catheter with transeptal approach was $1940 and $7466, respectively. Similarly, the typical actual cost of disposable supplies was also $1940 for right sided APs ablated using standard tools versus $4616 using 3D mapping and irrigated ablation catheters (Additional files 1, 2). Additionally, with longer times for set up and additional staffing costs using 3D mapping and transseptal approach for left sided APs, the differences in cost per case are even greater. When extrapolated to large volumes of ablation cases, potential cost savings are significant. Using standard approach without supplemental tools, long term success rate was 93.2% with initial procedure. 14 of 281 (5%) patients with failed initial ablations at our institution underwent repeat ablation. With repeat procedures using more supplementary tools, success rate was 100%. The cost savings of the initial procedures outweighs the additional cost of these 5% of cases requiring a second procedure. This study demonstrated that initial success rate and efficiency was lowest for AS and RFW pathways which is a small subset of all accessory APs. Initial use of 3D mapping and other advanced tools in these selective difficult cases, if AP location is known based on ECG localization of manifest pathways, may further improve cost effectiveness and efficiency. Overall, the tiered approach and selective use of advanced tools significantly reduces cost. In addition, the outcome data support that overall success, and safety are not compromised using this approach that maximizes resource utilization.

### Study limitations

AP locations were only documented as narratives in the procedure report with fluoroscopic images unavailable to verify a successful site. This could result in subjective inaccurate assignment of the pathway location. However, the inaccurate assignment would likely be to a close adjacent site and not across a major group.

Although specific reasons for prolonged procedure times were tabulated, their actual frequency of occurrence within a given location could not be quantified since only narrative information entered in the report at the discretion of the operator was included, with under-reporting a potential significant issue. Despite this, we were able the summarize common reasons for prolonged times within each specific site.

## Conclusions

Vast majority of accessory pathways are located in the left freewall and posteroseptal regions. Accessory pathways in these regions can be ablated with standard tools and approaches with high degree of efficiency and safety with excellent long-term outcomes. Right freewall, anteroseptal, left anterolateral, and coronary sinus accessory pathways are less common but more difficult to ablate as evidenced by relatively prolonged procedure time parameters and lower initial procedure acute success. We showed that within this large community-based referral practice, due to the high frequency of left freewall and posteroseptal pathways, a tiered approach strategy to AP ablation is effective and safe with potential for greater procedural and cost efficiency.

## Supplementary Information

**Additional file 1**. Disposable Supplies Cost Comparison. The additional table shows the comparison of typical actual cost of disposable supplies among procedures using standard tools for right and left sided APs with retrograde aortic access for left sided APs, versus using advanced tools including routine 3D mapping and transseptal access for left sided APs.

**Additional file 2**. Cost of Supplies for EP Cases KP 2020. The spreadsheet shows the vendors, part numbers, and cost of disposable supplies in 2021 US dollars.

## Data Availability

The vendor and costs (2021 US dollars) of disposable supplies for electrophysiology study and accessory pathway ablation procedures are included in supplemental materials. Datasets generated and/or analyzed during the current study are available from the corresponding author on reasonable request.

## Abbreviations

AP: Atrioventricular pathway
RF: Radiofrequency
SVT: Supraventricular tachycardia
AVRT: Atrioventricular reciprocating tachycardia
TVA: Tricuspid valve annulus
MVA: Mitral valve annulus
AS: Anteroseptal
PS: Posteroseptal
RFW: Right freewall
LFW: Left freewall
LPS: Left posteroseptal
RPS: Right posteroseptal
LPL: Left posterolateral
RPL: Right posterolateral
LAL: Left anterolateral
RAL: Right anterolateral
Epi-CS: Subepicardial coronary venous system
LAO: Left anterior oblique
