# Supplementary material for: Contemporary procedure characteristics and outcomes of accessory atrioventricular pathway ablations in an integrated community-based health care system using a tiered approach
Source: BMC Cardiovasc Disord. 2021 Jun 30;21:319. doi: 10.1186/s12872-021-02132-0 (PMC8243587; doi:10.1186/s12872-021-02132-0)
Supplement: Supplementary file 1 — Additional file 1. Disposable Supplies Cost Comparison. The additional table shows the comparison of typical actual cost of disposable supplies among procedures using standard tools for right and left sided APs with retrograde aortic access for left sided APs, versus using advanced tools including routine 3D mapping and transseptal access for left sided APs. [file 12872_2021_2132_MOESM1_ESM.docx]

Additional Supplemental Data:

**Disposable Supplies Cost Comparison**

Below is the comparison of typical actual cost of disposable supplies among procedures using standard tools for right and left sided APs with retrograde aortic access for left sided APs, versus using advanced tools including routine 3D mapping and transseptal access for left sided APs.

|  | Standard tools for right and left APs/ retrograde access for left AP | Transseptal access for left AP/ 3D mapping | 3D mapping for right AP |
| --- | --- | --- | --- |
| Duodecapolar catheter | 850 | 850 | 850 |
| Quadripolar catheters | 300 | 300 | 300 |
| Ablation kit | 20 | 20 | 20 |
| Femoral access sheaths | 40 | 40 | 40 |
| Sterile drape | 45 | 45 | 45 |
| Non-irrigated ablation catheter | 685 |  |  |
| Irrigated 3D enabled ablation catheter |  | 2964 | 2964 |
| Irrigation tubing |  | 60 | 60 |
| Long sheaths for transseptal access |  | 180 |  |
| Intracardiac echo catheter |  | 2450 |  |
| Transseptal needle |  | 220 |  |
| 3D mapping patches |  | 337 | 337 |
| Total Disposable Supplies Cost | **$1940** | **$7466** | **$4616** |
